# Semantic Processing Persists despite Anomalous Syntactic Category: ERP Evidence from Chinese Passive Sentences

**DOI:** 10.1371/journal.pone.0131936

**Published:** 2015-06-30

**Authors:** Yang Yang, Fuyun Wu, Xiaolin Zhou

**Affiliations:** 1 Institute of Linguistics, Shanghai International Studies University, Shanghai, 200083, China; 2 Center for Brain and Cognitive Sciences and Department of Psychology, Peking University, Beijing, 100871, China; 3 Key Laboratory of Machine Perception and Key Laboratory of Computational Linguistics (Ministry of Education), Peking University, Beijing, 100871, China; 4 PKU-IDG/McGovern Institute for Brain Research, Peking University, Beijing, 100871, China; University of Udine, ITALY

## Abstract

The syntax-first model and the parallel/interactive models make different predictions regarding whether syntactic category processing has a temporal and functional primacy over semantic processing. To further resolve this issue, an event-related potential experiment was conducted on 24 Chinese speakers reading Chinese passive sentences with the passive marker BEI (NP_1 _+ BEI + NP_2 _+ Verb). This construction was selected because it is the most-commonly used Chinese passive and very much resembles German passives, upon which the syntax-first hypothesis was primarily based. We manipulated semantic consistency (consistent vs. inconsistent) and syntactic category (noun vs. verb) of the critical verb, yielding four conditions: CORRECT (correct sentences), SEMANTIC (semantic anomaly), SYNTACTIC (syntactic category anomaly), and COMBINED (combined anomalies). Results showed both N400 and P600 effects for sentences with semantic anomaly, with syntactic category anomaly, or with combined anomalies. Converging with recent findings of Chinese ERP studies on various constructions, our study provides further evidence that syntactic category processing does not precede semantic processing in reading Chinese.

## Introduction

Syntactic and semantic processing are two crucial aspects of sentence comprehension, and an essential part of syntactic processing is the processing of syntactic category. There has been much debate regarding whether syntactic category processing temporally and functionally precedes semantic processing. Two major models have been proposed. The syntax-first approach claims the primacy of syntax (or syntactic category) over semantics [1–5]; in contrast, interactive models [6,7], including the constraint-based lexicalist/satisfaction model [8,9], the concurrent model [10], and the unification model [11], argue against the primacy of syntax.

Event-related potentials (ERPs) serve as an ideal measure to address this issue because several distinct ERP components are correlated with sentence comprehension. N400 is a negative potential that peaks approximately 400 ms after the target onset with a centro-parietal distribution [12] and sometimes with a more anterior distribution [13]. The N400 component has been seen as an electrophysiological sign of semantic anomaly indexing lexical-semantic integration difficulty [12]. Both early left anterior negativity (ELAN) effects and left anterior negativity (LAN) effects are considered to be related to syntactic processing difficulties [14–16], while the P600 effect, a centro-posteriorly distributed late positivity, is thought to reflect the reanalysis and repair processes in second-pass syntactic processing [17,18].

According to the syntax-first model [4], sentence parsing involves three stages: (i) an initial stage of structure building based on syntactic category processing (possibly an ELAN effect evoked by syntactic-category anomalies of critical words); (ii) syntactic processing (a LAN effect evoked by syntactic anomalies) and semantic processing such as thematic role assignment (an N400 effect elicited by semantic anomalies); (iii) a final stage of integrating syntactic and semantic information (a P600 effect elicited by syntactic anomalies).

In addition to the ELAN/LAN effect signifying the early stage of syntactic category-based syntactic processing, a number of ERP studies, particularly those on German passive sentences, have found the absence of the N400 effect in the case of combined semantic and syntactic-category anomalies as evidence for the syntax-first model [5,16,19–23]. The lack of N400 effect in the combined anomaly condition is interpreted as a failure of initial syntactic processing leading to a total block of semantic processing. This serves as evidence for the primacy of syntax over semantics, thus supporting the syntax-first model. Consider a German auditory experiment reported in Friederici et al. [22], where participants were asked to judge whether a word displayed on the screen had been heard in the previous sentence. In sentences with combined anomalies (e.g., *Das Buch wurde trotz* ***verpflanzt*** *von einem Verleger*, *den wenige empfahlen* ‘The book was despite **replanted** by a publisher whom few recommended’), only LAN-P600 effects were found after the onset of critical word (***verpflanzt***), with no trace of an N400 effect.

The absence of N400 effects in existing ERP studies on German passives is taken as solid proof for the syntax-first model. However, it is worth noting that German as a strong configurational language is rich in morpho-syntactic cues. Thus it is highly likely that the incorrect syntactic category deprives German comprehenders of useful cues to build up structures necessary for them to arrive at the meaning of the (passive) sentence.

Chinese can help to distinguish the two models because, as an isolating language, it has little morphology. Existing ERP studies on Chinese have tested a variety of syntactic constructions, including the BA construction [24–26], the canonical SVO sentence [26], the object-shift/topicalization construction [27], and the BEI construction [28]. An emerging pattern arising from these studies is that an N400 effect *can* be found in the combined anomaly condition, in contrast to ERP results from German. The presence of the N400 effect seems to suggest that syntactic category processing is not a prerequisite of semantic integration, at least for Chinese.

However, these studies may not constitute a solid counterargument to German ERP work due to (i) lack of cross-linguistic comparability, (ii) availability of alternative accounts, and (iii) controversy over specific manipulations. We review each aspect below.

First, not all syntactic structures in Chinese have fitting counterparts in German, making direct comparisons rather difficult. For instance, the BA construction (S-BA-O-V) is a language-specific structure, in which the object noun phrase (NP) or the theme/patient occurs after the marker BA but before the verb. This construction is perhaps the most tested structure thus far in Chinese ERP studies. Similarly, the object shift or topicalization structure (OSV) is widely used in Chinese, but much less so in German unless licensed by special discourse contexts. Thus, any differences in EEG patterns associated with combined anomalies in Chinese might simply be due to some idiosyncrasies of Chinese grammar, rather than reflecting the predictions of the two parsing models.

Second, results of some Chinese ERP studies might be subject to different interpretations. For instance, in an auditory ERP study on the BA construction, Ye et al. [24] created syntactic-category anomaly by deleting the object NP after BA and semantic anomaly by violating the semantic selection of the verb, as in the combined anomalies (e.g., 伐木工开采森林, 把**裁**了‘Exploiting the forest, the lumberjack BA (pine trees) **cut**’). They found broad negativities in the 300–500 ms range (N400) after the onset of the critical verb. But given that the critical verb occurred at the end of the sentence, such negativities could be related to sentence-final wrap-up processes [29]. Furthermore, Chinese is a tonal language and has many homophones, which means that different words share the same pronunciation. Given the auditory modality used in this study, the negativities could be partly due to semantic confusion caused by homophone activation.

Third, in terms of ERP experimental design, the criterion for syntactic category violation is less straightforward in Chinese than in strong configurational languages such as German. This applies to several studies including Wang et al. [28] on the Chinese passive BEI construction, where the theme/patient precedes BEI, and the agent occurs after BEI. They created combined anomalies by using an intransitive verb (e.g. ‘sobbed’), which violated both (sub)categorization selection and semantic selection (given the preceding agent NP), as in 失踪儿童被不法分子**啜泣**到了山区 ‘The lost children were **sobbed** to mountain areas by outlaws’. The assumption here was that incorrect verb transitivity (verb-subcategorization violation) involves both syntactic violation and semantic mismatch. Wang et al. [28] observed an N400-P600 pattern in this condition. However, one might argue that this verb-subcategorization violation should not, in a strict sense, be considered as syntactic category violation, and hence the pattern of effect should not be taken as evidence against the syntax-first view. Indeed, this N400-P600 response for subcategorization violation has been demonstrated in several previous studies, including the study by Friederici herself [20,30].

Leaving aside the problematic manipulation of verb (in)transitivity, Zhang and colleagues’ work on the BA construction appears to be especially enlightening [25,26]. In Experiment 1 of Zhang et al. [26], they created combined anomalies by replacing the post-adverbial verb (e.g., ‘peel’) with a noun (e.g., ‘piano’), thereby violating both word category and semantic selection of a verb that is highly expected (given the syntactic frame of the BA construction and the preceding adverb ‘slowly’), as in 李薇把新鲜的鸭梨慢慢地**钢琴**了两个 (*Li Wei BA fresh pears slowly* ***piano-*** *le*_*PERF*_ *two*, ‘Li Wei piano-ed two fresh pears slowly’). The N400 effect was found in combined anomalies, indicating that semantic integration persisted even when syntactic category-based structure building presumably failed. Yet given that the BA construction tested in Zhang et al. is specific to Chinese, their conclusion might not be generalized unless further evidence is obtained using some construction commonly shared between Chinese and German.

In the current study, we aimed to overcome the above difficulties, including the ‘comparability’ problem, by focusing on Chinese passive sentences, which structurally resemble German passives. Both languages use the general format of *Patient NP + BEI/Von + Agent NP + VP* to express ‘That piece of glass is carefully **wiped** by Jiangna’, as shown by the following grammatical Chinese passive sentence (1a) and its German counterpart (1b):

1.
  - nakuai boli bei Jiangna zixide **cashil**e. that-CL glass BEI Jiangna carefully **wipe**-ASP.
  - Jene Scheibe wurde von Jiangna sorgfältig **gewischt**. that glass by Jiangna carefully **wiped**.

We adopted the same design as Zhang et al. [26] on the BA construction by manipulating semantic consistency (consistent vs. inconsistent) and syntactic category (noun vs. verb) of the critical verb. We hypothesized that semantic anomaly would elicit an N400 effect, and syntactic-category anomaly would elicit a P600 effect. Critically, for combined anomalies, there would be no N400 effects according to the syntax-first model, but according to interactive/concurrent models, it would result in a strong N400 effect.

## Method

### Participants

Twenty-eight right-handed undergraduate and graduate native Chinese speakers from Peking University or neighboring universities were paid to participate in the experiment. Four were excluded from data analysis due to excessive eye or head movements artifacts (over 40% trials). The remaining 24 participants (13 female) aged between 19 and 25 years, with a mean age of 21.6 (SD = 1.86) years. No participants reported any cognitive or psychiatric disorders or vision deficit (after correction). Informed written consent was obtained from each participant before the test. This study was carried out in accordance with the Declaration of Helsinki and was approved by the Ethics Committee of the Department of Psychology, Peking University.

### Materials and norming tests

In our study, the structure of the passive stimuli is “Det + NP1 (inanimate) + BEI + NP2 (animate) +ADV + *V/N* + Le (PERF) + FreqAdv”. We added an aspect marker-*le* and an adverbial modifier after the critical verb for two reasons. First, sentences can continue after the critical verb, allowing us to avoid the sentence-final wrap-up effects. Second, Chinese passive structures typically denote completion of an action [31,32], thus an aspect marker and a frequency-denoting adverbial modifier can convey a sense of completion associated with the verbs. All two-character human names serving as agent NPs are taken from Jiang and Zhou [33], with word frequencies and number of strokes well controlled across conditions.

All sentences were visually presented segment by segment (see Table 1, the word between two slashes presented as one segment in one screen). We manipulated the verb’s semantic consistency (SEM) to its argument/patient NP (consistent vs. inconsistent) and its syntactic category (SYN) (verb vs. noun), yielding four conditions in Table 1: CORRECT (SEM+, SYN+), SEMANTIC (SEM-, SYN+), SYNTACTIC (SEM+, SYN-), and COMBINED (SEM-, SYN-).

**Table 1:** Exemplar stimuli for the four critical conditions, with English translations.

**a**. CORRECT	那块/玻璃/被/蒋娜/仔细地/**擦拭**/了多遍。
	Det/glass/ BEI/ Name/carefully/***wipe***/ASP/many times.
	(That piece of glass is carefully ***wiped*** by Na Jiang many times.)
**b**. SEMANTIC	那个/方案/被/胡杰/仔细地/**擦拭** */*了/多遍。
	Det/plan/ BEI/ Name/carefully/***wipe***/ASP/many times.
	(That plan is carefully ***wiped*** by Jie Hu many times.)
**c.** SYNTACTIC	那块/玻璃/被/蒋娜/仔细地/**抹布** */*了/多遍。
	Det/glass/ BEI/ Name/carefully/***dishcloth***/ASP/many times.
	(That piece of glass is carefully ***dishcloth*** by Na Jiang many times.)
**d**. COMBINED	那个/方案/被/胡杰/仔细地/**抹布** */*了/多遍。
	Det/plan/ BEI/ Name/carefully/***dishcloth***/ASP/many times.
	(That plan is carefully ***dishcloth*** by Jie Hu many times.)

The critical words are in bold.

#### Five Norming pretests

We conducted five pretests to check various properties of the stimuli. The first test was conducted on all syntactic-category anomalous sentences (in the SYNTACTIC and COMBINED conditions), in which the critical region was a noun. A group of 20 participants who did not participate in the ERP test were asked to make the sentences more natural or acceptable by changing whichever word(s) they thought necessary. On average, 99% of the time participants correctly identified the anomalous nouns, and changed them to transitive verbs. These results showed that participants detected the syntactic-category anomaly at the critical region and expected the words to be verbs.

To further quantify the degree of semantic anomaly across conditions, we conducted a second pretest on comprehensibility/semantic acceptability of the critical sentences. A different group of 32 participants were asked to judge the comprehensibility of each sentence on a 5-point scale, with 1 meaning completely incomprehensible and 5 quite comprehensible. Table 2 shows the mean ratings in the four conditions. A repeated measures ANOVA with semantic consistency and syntactic category as two within-participant factors showed a significant effect of semantic consistency, F(1, 31) = 662.64, p < 0.001, a significant main effect of syntactic category, F(1, 31) = 143.42, p < 0.001, and a significant interaction between them, F(1, 31) = 57.92, p < 0.01. Further analysis showed that the comprehensibility scores differed between conditions, even for the smallest difference between SEMANTIC and COMBINED conditions, F (1, 31) = 41.14, p < 0.001.

**Table 2:** Mean scores of sentence comprehensibility rating (on a 5-point scale), cloze probability for the critical words, the semantic relatedness between the critical words, the mostly produced words in the cloze probability test (on a 7-point scale) and the plausibility of the construction (standard deviations in parentheses).

	CORRECT	SEMANTIC	SYNTACTIC	COMBINED
Comprehensibility	4.69 (0.29)	1.92 (0.52)	3.30 (0.50)	1.64 (0.38)
Cloze probability	21%	0%	0%	0%
Semantic relatedness	4.76 (0.19)	2.40 (0.18)	4.12 (0.23)	2.20 (0.16)
Plausibility of construction	4.57 (0.08)	4.57 (0.08)	1.97 (0.11)	1.97 (0.11)

The third pretest was on cloze probability of the critical verbs. Another group of 20 participants were asked to complete sentence fragments “Det + NP1 (inanimate) + BEI + NP2 (animate) + ADV …” with the first appropriate words coming into their minds. Results showed that while the verbs used in the CORRECT condition had a cloze probability of 21%, critical words in the other three conditions all had a cloze probability of zero.

The fourth pretest was on semantic relatedness between the critical words used in each condition and the words with the highest production rate in the above cloze probability test. The purpose of this pretest was to provide evidence for an account of N400 effects in different conditions (See Discussion). Twenty-two participants were asked to rate on a 7-point scale (1 = completely unrelated, 7 = most highly related) the semantic relatedness between the critical words and the produced words. The mean rating scores for the four conditions are presented in Table 2. A repeated-measures ANOVA with semantic consistency and word category as two within-participant factors showed a significant main effect of semantic consistency F(1, 21) = 322.00, p < 0.001, a significant main effect of word category, F(1, 21) = 31.54, p < 0.001, and a significant interaction between them, F(1, 21) = 5.72, p < 0.05. Further tests showed that while the small difference between CORRECT and SYNTACTIC reached significance, F(1, 21) = 18.86, p < 0.001, so did the smallest difference between SEMANTIC and COMBINED, F(1, 21) = 6.80, p < 0.05.

To further determine the validity of our stimuli, we conducted the fifth pretest on sentence congruency related to our phrasal structure, in order to make sure that the transitive verbs could indeed be used in the BEI constructions, and that the counterpart nouns used in the SYNTACTIC condition really did not make sense in such structures. The phrasal segments (e.g., “被…仔细地擦拭”, *BEI…carefully wipe*) were rated on a 5-point scale by another group of 34 participants regarding how plausible it was to use the phrasal segments to construct a congruent sentence (ranging from 1 = extremely implausible to 5 = fully plausible). A repeated measures ANOVA revealed a significant main effect of condition, F (1, 33) = 219.30, *p* < 0.001. Post-hoc Bonferroni comparisons showed that the mean score of the noun in SYNTACTIC and COMBINED (mean = 1.97, SD = 0.11) was significantly lower than that of the transitive verbs in CORRECT and SEMANTIC (mean = 4.57, SD = 0.08).

#### Materials

Each participant read 160 critical sentences, with 40 in each condition. Word frequencies and strokes of critical words and Patient NPs were well matched across conditions (*p* > .1). In addition, 220 filler items were used to prevent participants from developing test-taking strategies. Eighty were correct BEI sentences with two NPs varied in their animacy status. These sentences were included to equate the numbers of the correct and incorrect BEI sentences overall and to offset the inanimate-animate configuration used in the critical sentences. The remaining 140 fillers were of different syntactic structures, including the BA construction, simple SVO sentences, topicalization, and complex clauses.

Four lists were created using a Latin Square design. In each list, 160 critical sentences and 220 fillers were pseudo-randomized, such that no more than 4 BEI sentences and no more than two critical sentences of the same condition appeared consecutively.

### Procedure

Participants sat approximately 100 cm away from a CRT computer screen in a dim and sound-proofing room. They were instructed to move their head and body as little as possible and to keep their eyes fixated on a cross sign at the center of the computer screen before the onset of each sentence. Sentences were presented in white against black background segment by segment. Each segment was presented for 400 ms followed by a blank screen lasting 400 ms. Trials were separated by a 2000 ms interval.

Participants were instructed to read all trials attentively for comprehension. About one third of the trials (80 critical sentences and 50 fillers) were followed by a cue “?”, upon which participants need to judge the correctness of the sentence by pressing one of the two response buttons. The cue remained on the screen for a maximum of 3000 ms until participants responded. The cues were pseudorandomized such that 1) at least one cue appeared in every 5 consecutive sentences; 2) for answers, at most three “Yes” or “No” occurred consecutively (if responded correctly).

Trials were presented in five blocks, each with 76 sentences. Participants could take a short break between blocks. Prior to the formal test, each participant received 25 separate sentences for practice. The whole experiment lasted about 2 hours including electrode preparation.

### ERP recording

Continuous EEGs were recorded from 62 electrodes in a secured elastic cap (Electrocap International) localized at the following sites: AF7, AF3, FP1, FPZ, FP2, AF4, AF8, F7, F5, F3, F1, Fz, F2, F4, F6, F8, FT7, FC5, FC3, FC1, FCZ, FC2, FC4, FC6, FT8, T7, C5, C3, C1, CZ, C2, C4, C6, T8, TP7, CP5, CP3, CP1, CPZ, CP2, CP4, CP6, TP8, P7, P5, P3, P1, PZ, P2, P4, P6, P8, PO7, PO5, PO3, POZ, PO4, PO6, PO8, O1, Oz and O2. EEGs on these electrodes were referenced online to the tip of nose and were re-referenced offline to the mean of the left and right mastoids. The vertical electro-oculogram (VEOG) was recorded from electrodes placed above the right eye. The horizontal EOG (HEOG) was recorded from electrodes placed at the outer cantus of left eye. Electrode impedance was kept below 5 kΩ. The biosignals were amplified with a band pass from 0.016 to 100 Hz and digitized on-line with a sampling frequency of 500 Hz. ERPs were additionally filtered for plots with 20Hz low pass. The ocular artifacts were corrected automatically, with both VEOG and HEOG as common reference and blink detection by algorithms implemented in Brain Vision Analyzer. The original ERP data and the data produced during the analysis can be found in Harvard Dataverse Database (doi:10.7910/DVN/28781).

### ERP analysis

ERPs were computed for each sentence type, electrode site, and participant. Sentences contaminated by excessive movement artifacts (mean voltage exceeding ±100 μV) or incorrectly judged were excluded before averaging. The overall trials rejection rate was 11.3% across all 24 participants and conditions. The mean rejection rate for each condition was 13.3% (SD = 0.14) for CORRECT, 12.3% for SEMANTIC (SD = 0.14), 9.2% (SD = 0.09) for SYNTACTIC and 10.5% (SD = 0.14) for COMBINED.

Analyses were based on the critical verbs in the critical sentences. Since the critical words were preceded by different words in different conditions (see Table 1), we used a post stimulus-onset baseline covering 100 ms post critical-word-onset, following Friederici et al. [22]. Subsequent analyses were based on 800 ms-epochs post onset of the critical words. Two time windows were chosen on the basis of visual inspection and previous studies [15] [25]: (1) 300–500 ms time window for possible N400 effects; (2) 500–800 ms for possible P600 effects. A repeated measures analyses of variance (ANOVAs) were conducted on the average ERP amplitudes in the two time windows with following within-subjects factors: semantic consistency (SEM+, SEM-), syntactic category (SYN+, SYN-), hemisphere (left, middle and right) and region (anterior, central and posterior). Crossing the factors of hemisphere and region produced nine regions of interest (ROI), each with 6, 4, or 2 electrodes, including left anterior (F1, F3, F5, FC1, FC3, FC5), left central (C1, C3, C5, CP1, CP3, CP5), left posterior (P1, P3, P5, PO3), middle anterior (FZ, FCZ), middle central (CZ, CPZ), middle posterior (PZ, POZ), right anterior (F2, F4, F6, FC2, FC4, FC6), right central (C2, C4, C6, CP2, CP4, CP6) and right posterior (P2, P4, P6, PO4). Mean amplitudes were averaged over electrodes in each ROI for statistical purpose. Comparisons were planned for each ROI if interactions reached significance. The Greenhouse-Geisser correction was applied when the evaluated effects had more than one degree of freedom in the numerator. For planned comparisons, the probability levels were Bonferroni-adjusted.

## Results

### Behavior results

The overall response accuracy rate was 91.6% across all four conditions: 87.7% for the correct sentences (SD = 0.1); 89.2% for the semantic anomaly condition (SD = 0.1); 93.8% for the syntactic-category anomaly condition (SD = 0.09); 96% for the combined anomalies (SD = 0.06). A repeated-measures ANOVA with semantic consistency and word category as two within-participant factors showed only a significant main effect of syntactic category, F (1, 23) = 14.59, p < 0.01, with the accuracy higher for sentences in the SYNTACTIC and COMBINED conditions than for sentences in the CORRECT and SEMANTIC conditions. We did not measure RTs in this study, following Friederici et al. [22] and Zhang et al. [26]. RTs were not informative in the current study as they were recorded long after the presentation of each sentence. In general, behavioral results showed that participants were attentive to the task.

### ERP data

**Fig 1:**
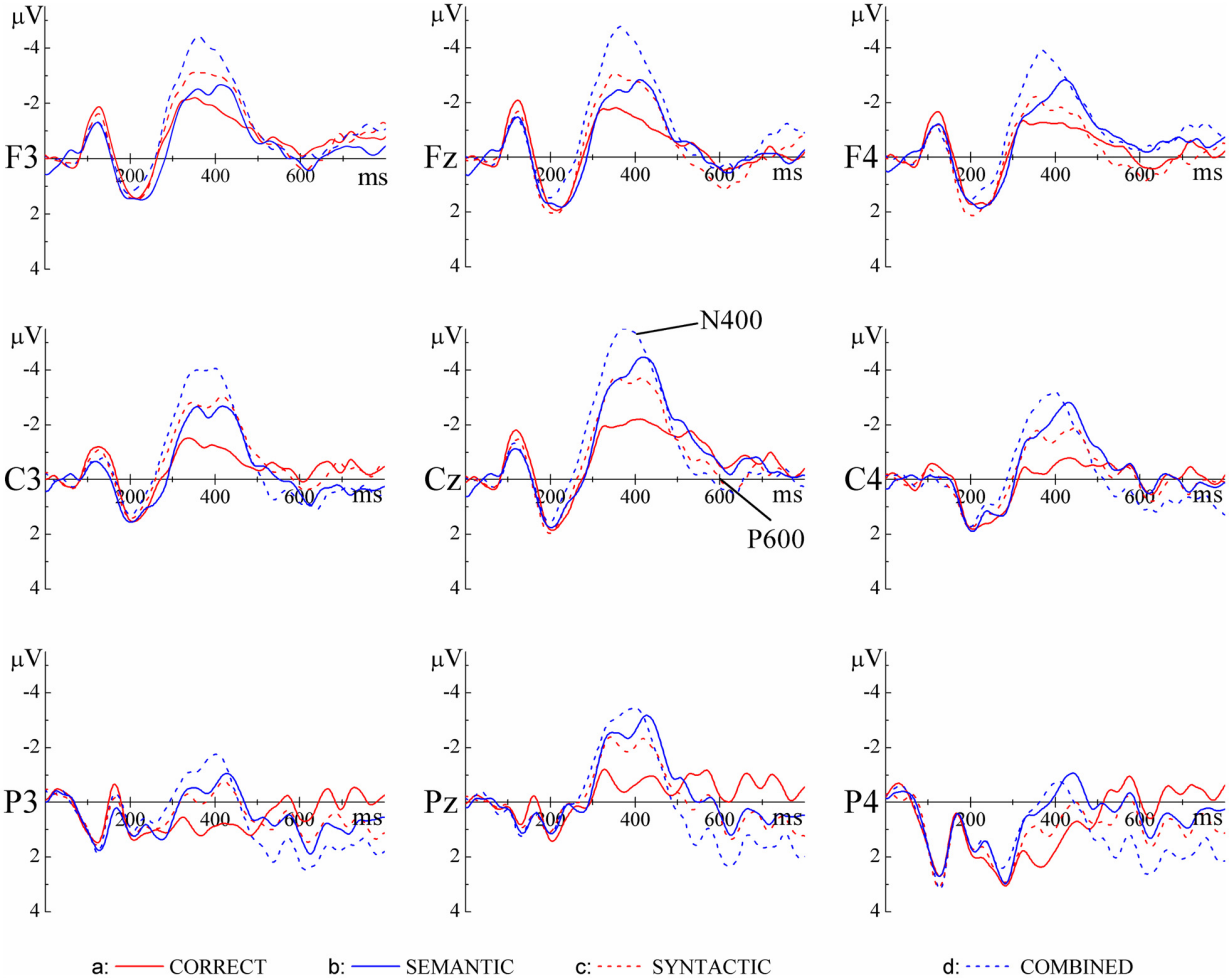
Grand average ERPs at 9 exemplar electrodes time-locked to the onset of the critical words for the four experimental conditions.

**Fig 2:**
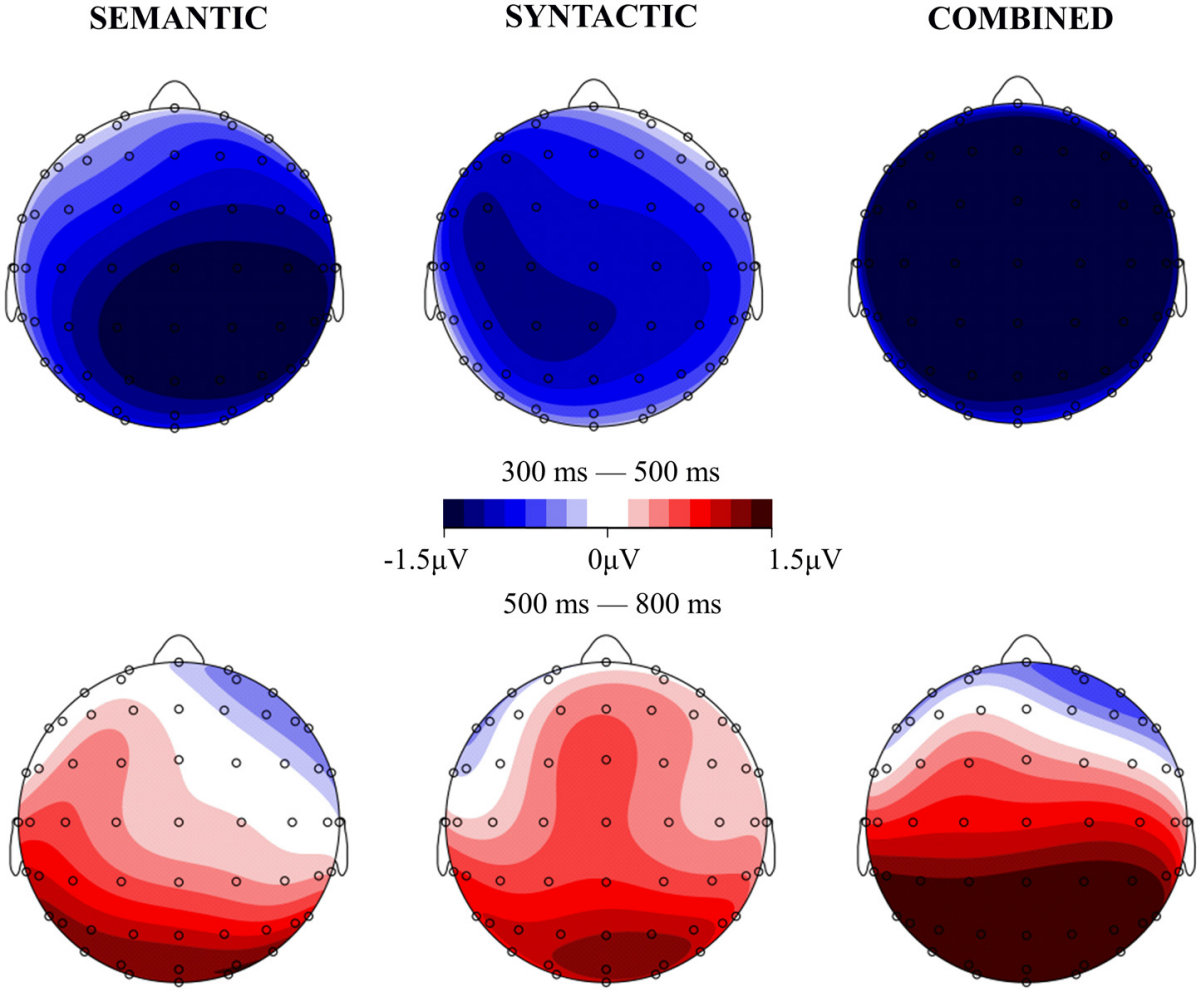
Topographic distributions of the mean ERP differences at the 300–500 ms and 500–800 ms windows, respectively. The three anomalous conditions were all compared with the CORRECT condition.

As shown in Fig 1 and Fig 2, in the 300–500 ms time window, compared with the CORRECT sentences, anomalous sentences in all the other three conditions (SYNTACTIC, SEMANTIC and COMBINED) elicited larger negativities (N400 effects). These effects had somewhat different distributions over the scalp (Fig 2), with the effect for SYNTACTIC predominantly on the left hemisphere and the effect for COMBINED over the whole scalp. In the time window of 500–800 ms, compared with the CORRECT sentences, sentences in the SYNTACTIC and COMBINED conditions elicited larger positivities (P600) in the centro-posterior areas whereas the effect for the SEMANTIC conditions was more left-lateralized. Statistical analyses confirmed these observations.

#### The 300–500 ms time window

A repeated-measures ANOVA revealed a main effect of semantic consistency, F(1, 23) = 22.2, *p* < 0.01, suggesting that sentences in the SEMANTIC and COMBINED conditions evoked a larger N400 than sentences in the CORRECT and SYNTACTIC conditions. This effect did not interact with syntactic category, F(1, 23) = 0.341, *p* > 0.1, but interacted with hemisphere and region, F(4, 92) = 4.167, *p* < 0.01, indicating that the size of the main effect of semantic consistency varied over different scalp areas (Fig 2). Importantly, the main effect of syntactic category also reached significance, F(1, 23) = 16.41, p < 0.05, with sentences in the SYNTACTIC and COMBINED conditions evoking more negative responses than sentences in the CORRECT and SEMANTIC conditions. This effect interacted with hemisphere and region, F(4, 92) = 2.31, 0.05 < p < 0.1, indicating that the size of this varied over different scalp areas.

To better understand the N400 effects for the three types of anomalous sentences, we conducted separate comparisons between the SYNTACTIC, SEMANTIC and COMBINED conditions and the CORRECT condition. Compared with the sentences in the CORRECT condition, sentences in the SEMANTIC condition elicited more negative responses, F(1, 23) = 10.11, p < 0.01, and this N400 effect interacted with hemispheres, F(2, 46) = 5.06, p = 0.01 and region, F(2, 46) = 3.35, p < 0.05. It is clear from Fig 2 that the N400 effect was the strongest in the right centro-posterior regions. Sentences in the SYNTACTIC condition also elicited more negative responses, F(1, 23) = 6.96, p < 0.05. Moreover, sentences in the COMBINED condition elicited the most negative responses, F(1, 23) = 33.753, p < 0.001, with the effect appearing all over the scalp sites (Fig 2).

Given that the N400 effect appeared in all three anomalous conditions, we further examined the relative magnitude of the effect in each condition. A repeated-measures ANOVA was conducted on the ERP responses in the three anomalous conditions after subtracting the ERP responses in the CORRECT condition. The difference between the effects for the SEMANTIC and SYNTACTIC conditions did not reach significance, F(1, 23) = 1.17, p > 0.1, although it did interact with hemisphere and region, F(4, 92) = 2.46, p = 0.05. Detailed analyses for ROIs showed that the N400 effect was stronger for the SEMANTIC condition than for the SYNTACTIC condition in middle posterior, right central and right posterior areas (p < 0.05 or 0.05 < p < 0.1). Importantly, the N400 effect for the COMBINED condition was larger than the effect for either the SEMANTIC or SYNTACTIC conditions: F(1, 23) = 3.19, 0.05 < p < 0.1, and F(1, 23) = 6.74, p < 0.05, respectively (Fig 2).

#### The 500–800 ms time window

A repeated-measures ANOVA revealed a significant main effect of syntactic category, F(1, 23) = 8.178, *p* < 0.01, with syntactically anomalous sentences in the SYNTACTIC and COMBINED conditions being more positive than sentences in the CORRECT and SEMANTIC conditions. This effect interacted with hemisphere, F(2, 46) = 3.29, *p* < 0.05, and region, F(2, 46) = 10.2, *p* < 0.01, respectively. Separate analyses were conducted for each ROI. The syntactic category effect reached significance in all centro-posterior ROIs, including left central, left posterior, middle central, middle posterior, right central and right posterior, Fs(1, 23) > 4.44, *ps* < 0.05.

Importantly, although the main effect of semantic consistency was not significant in this window, F(1, 23) = 1.913, *p* > 0.1, it interacted with hemisphere and region, F(4, 92) = 3.09, *p* < 0.05. Detailed analyses in each ROI found an overall semantic consistency effect in left posterior, middle posterior and right posterior regions, Fs(1, 23) > 6.8, ps < 0.05.

To better understand the P600 effects for the anomalous sentences, we conducted a further analysis comparing the SYNTACTIC, SEMANTIC and COMBINED conditions separately with the CORRECT condition. Results showed that, compared with the CORRECT condition, P600 in the SEMANTIC condition did not show a significant main effect, F(1, 23) = 1.47, p > 0.1, but it interacted marginally with hemisphere, F(2, 46) = 2.52, 0.05 < p < 0.1, and more strongly with region, F(2, 46) = 8.42, p < 0.01. Detailed analysis in each ROI showed effects in three posterior ROIs were significant (ps < 0.05). It is clear from Fig 2 that the P600 effect for the SEMANTIC condition was somewhat lateralized to the left posterior regions. Similarly, compared with the CORRECT condition, sentences in the SYNTACTIC condition did not show a significant main effect of P600 either, F(1, 23) = 2.01, p > 0.1, but this effect interacted with region, F(2, 46) = 4.59, p < 0.05. Detailed analysis showed a significant effect in the posterior areas (ps < 0.05). Finally, compared with the CORRECT condition, sentences in the COMBINED condition evoked stronger positivities, F(1, 23) = 5.97, p < 0.05. This effect also interacted with region, F(2, 46) = 23.20, p < 0.01. Detailed analysis showed that this P600 effect was significant in both the central and the posterior areas (p < 0.05 or p < 0.01). Thus, compared with the CORRECT condition, sentences in all three anomalous conditions elicited P600 effects, although the distribution of the effect varied over conditions (Fig 2).

We compared the P600 effects between conditions. The difference between SEMANTIC and SYNTACTIC was not significant, F(1, 23) = 0.25, p > 0.1; however, it interacted with hemisphere and region, F(4, 92) = 3.31, p < 0.05. It is clear from Fig 2 that the P600 effect for the SEMANTIC condition was more left-lateralized while the P600 effect for the SYNTACTIC condition was more centro-posteriorly distributed. Moreover, the P600 effect for the COMBINED condition did not differ from the effect for the SEMANTIC condition, F(1, 23) = 1.19, p > 0.1, or from the effect for the SYNTACTIC condition, F(1, 23) = 0.33, p > 0.1, although the differences between conditions interacted with region (ps < 0.05), indicating that the P600 effects in the three anomalous conditions were differentially distributed over the scalp.

## Discussion

The main goal of this study was to test whether Chinese sentence processing was consistent with the syntax-first model by using the BEI construction, a passive structure that has not been extensively studied but is most comparable to the German passive. Overall, we found both N400 and P600 effects for sentences with semantic anomaly, with syntactic category anomaly, or with combined anomalies. Our results replicate and extend findings from previous Chinese ERP studies that used different structures [24–27] and present a solid piece of evidence against the syntax-first model.

The present findings demonstrate the importance of probing processing differences from a cross-linguistic perspective. By using passive sentences, we ruled out the potential confound of sentence structure in the previous ERP studies on Chinese. As stated in the introduction, evidence supporting the syntax-first model comes mainly from German passive structures [15,16,22]. However, existing ERP results in Chinese that conflict with this model could be attributed to the idiosyncrasies of the Chinese language. By using the Chinese BEI structure that closely resembles the German passive structure, we obtained a pattern of effects that supports existing ERP work on the BA construction and offers converging evidence that semantic processing in Chinese does not need to be licensed by syntax.

After controlling for structural differences between the two languages, what is the real reason behind the fact that Chinese ERP studies are inconsistent with the syntax-first model? As Zhou, Ye, Cheung and Chen [34] stated, it is likely that there exist language-specific cognitive processes. Thus, one possibility is that processing models may take into account and incorporate typological differences between languages. Consider German again. As a strong configurational language, its rich morphological cues (e.g. inflectional affixes) explicitly mark different syntactic information such as tense, case, and number. Moreover, syntactic categories such as nouns (which are capitalized in written German) or verbs are easy to visually identify by their morphological forms. Therefore, it is possible that syntactic structure building based on syntactic categories is fast and even automatic. In contrast, Chinese is an isolating language that is impoverished in morphological inflections. Often, comprehenders cannot detect the syntactic category of a Chinese word simply by its form, and the boundaries between nouns and verbs as syntactic classes in Chinese are by no means distinct [35,36]. Without explicit grammatical cues, syntactic structure building for a Chinese sentence relies mainly on the processing of the lexical and contextual meaning of each individual word [28]. Therefore, it seems plausible that semantic information, rather than syntactic information, has primacy in Chinese. As stated in Kuperberg [37], a semantic memory-based analysis and possibly a semantically-driven combinatorial thematic analysis can temporarily dominate online sentence comprehension.

Four findings in the current study are particularly worth discussing. First, as shown in Fig 1, in the 300–500 ms time window, responses to sentences with combined anomalies were more negative than responses to sentences with only a semantic anomaly or only a syntactic anomaly. This was consistent with the rating test in which sentences with combined anomalies were rated as more difficult to comprehend than sentences with single anomalies (Table 2). As discussed in the introduction, the absence of N400 effects in the COMBINED condition is taken as evidence for the syntax-first model, because the unsuccessful syntactic processing entirely blocks access to lexical/semantic processing. However, in our study, we found N400 effects in the COMBINED condition, leading us to conclude, reflecting Zhang et al. (2010), that syntactic category violation does not prevent access to lexical semantics of the target word; but it would make the semantic integration of this word into the sentence context more difficult. Another source of increased N400 responses in the COMBINED condition could come from the semantic relatedness between the critical words and the words that would be expected in the context (although these words were not presented). Federmeier and Kutas [38] demonstrated that words that were expected with respect to sentential context but were from the same semantic category as the expected words elicit reduced N400 responses, compared with words that were expected and were from different categories. This finding was interpreted as reflecting the impact of context-independent long-term memory structure on sentence processing: semantic features shared between the target words and the unexpected but related words are activated by sentence context, facilitating to a certain degree the integration of the former with the context. For the present study, although the critical words and the most expected words were largely from the same category (verbs for the SEMANTIC condition) or from different categories (nouns vs. verbs for the SYNTACTIC and COMBINED conditions), they nevertheless varied over conditions in terms of semantic relatedness (Table 2). It is possible that this variation contributed to the differential N400 responses in the three anomalous conditions.

Second, compared with the correct sentences, sentences with syntactic anomaly also elicited increased N400 responses. This effect seems surprising. But both comprehensibility ratings and the rating of semantic relatedness between the critical words and contextually-expected words (Table 2) indicated that participants had more difficulties in integrating the critical nouns, which violated the expectancy of verbs at the critical position in the SYNTACTIC condition, compared with the processing of critical verbs in the CORRECT condition. The finding of increased N400 responses for the SYNTACTIC condition, as compared with the CORRECT condition, was very much consistent with the finding of increased N400 responses for the COMBINED condition, as assessed against the SYNTACTIC and SEMANTIC conditions.

The third noteworthy finding is that in the 500–800 ms time window, we observed a positivity effect for sentences with semantic anomaly in a parietal region. This effect appeared to be similar to the “semantic P600” that was reported not only for thematic role reversals [28,39–41], but also for the violations of semantic constraints between the verb and the object noun in a complex syntactic structure [42,43]. Jiang and Zhou [43] suggested that the appearance of the (left-lateralized) semantic P600 indicates the initiation of a coordination process for multiple semantic processes at different levels of syntactic hierarchy. When the semantic process at one level encounters difficulties, the processing system may initiate a process redeploying the processing focus from this level to the semantic process at another level, in order to mitigate the difficulty in constructing a meaningful representation. Differing from the previous studies in which the semantic P600 effects were observed at the position of object nouns, our P600 effect here was obtained on the verbs, which were also embedded in a hierarchical structure. It is plausible that such coordination was also initiated by the input of the incorrect verb which had to satisfy both the local constraints between the adverbs and the verb and the long-distance dependency between the object noun and the verb. Further studies are needed to verify our findings and to choose between different accounts of the semantic P600.

The fourth noteworthy finding in our study is the “asymmetry” between semantic and syntactic processing. During the 300-500ms time window, we found a significantly stronger N400 effect in the COMBINED condition than in the SEMANTIC condition; however, we find no difference for the P600 effect between the SYNTACTIC and COMBINED conditions. This asymmetry seems consistent with the findings of Hagoort (2003), who specifically tested the effects of combined violations in relation to the effects of single semantic and single syntactic violations in Dutch. As suggested by Hagoort (2003), semantic integration is influenced by syntactic processing, however, the assignment of syntactic structure is independent of semantic context. We are cautious about whether our study supports this conclusion, because (i) a number of studies have also shown an increased P600 in the COMBINED condition relative to the SYNTACTIC condition (Friederici et al., 2004; Wang et al., 2013) and (ii) the increased N400 effect in the COMBINED condition of our study can be largely explained by the results of pre-tests and the negativities in the SYNTACTIC condition (see above discussion). It is possible that the interplay of syntax and semantics is asymmetric during online processing, but more work is needed in order to fully understand the underlying mechanism.

To conclude, the current study on Chinese passive sentences is consistent with studies on other Chinese structures [24–28] and demonstrates an N400 effect for sentences with both semantic anomaly and syntactic category anomaly, indicating that semantic processing persists in face of anomalous syntactic structure. Claims of syntactic category processing primacy do not apply to Chinese.
